# Renal involvement in hypereosinophilic syndrome: a systematic review with narrative synthesis of pathology, diagnosis, and therapy

**DOI:** 10.3389/fimmu.2026.1820583

**Published:** 2026-04-23

**Authors:** Qi-shun Wu, Zhi-liang Yu, Xin Lin, Zhang-li Wu, Ling Yang

**Affiliations:** Division of Nephrology, Department of Medicine, The Second Affiliated Hospital of Wannan Medical University, Wuhu, Anhui, China

**Keywords:** eosinophilic interstitial nephritis, hypereosinophilic syndrome, IgG4-related disease, kidney disease, narrative synthesis

## Abstract

**Background:**

Renal involvement in hypereosinophilic syndrome (HES) occurs in 5-10% of patients but remains poorly characterized. Diagnostic overlap with IgG4-related kidney disease (IgG4-RKD) presents clinical challenges.

**Objective:**

To characterize the clinicopathologic spectrum of histologically confirmed HES-related kidney disease, examine diagnostic differentiation from IgG4-RKD, and appraise therapeutic evidence.

**Methods:**

Systematic review with narrative synthesis following PROSPERO-registered protocol (CRD420261307200). Six databases were searched from inception to February 27, 2026. Dual independent screening and domain-specific GRADE assessment were performed. Due to uncontrolled study designs and high heterogeneity, narrative synthesis was conducted rather than quantitative meta-analysis.

**Results:**

From 2, 247 records, 37 studies (42 cases) met inclusion criteria. Acute eosinophilic interstitial nephritis predominated (54.8%, 23/42), followed by thrombotic microangiopathy (19.0%, 8/42), chronic interstitial fibrosis (11.9%, 5/42), and glomerular lesions (14.3%, 6/42). Nine cases (21.4%) exhibited IgG4-RKD-like features with IgG4+/IgG+ ratios below 40% (median 21%, IQR 14-30%). However, substantial technical heterogeneity limits clinical applicability. Fourteen cases received anti-IL-5/IL-5Rα biologics; all reported improvement with no documented failures, suggesting publication bias. No randomized controlled trials exist for HES-related kidney disease.

**Certainty of evidence:**

Descriptive pathology findings were rated low certainty (downgraded for selection bias from biopsy requirement). IgG4/IgG ratio observations were very low certainty (downgraded for risk of bias, inconsistency, and indirectness). Treatment efficacy conclusions were very low certainty (downgraded for all GRADE domains, with publication bias and indirectness being most critical).

**Conclusion:**

This systematic review provides hypothesis-generating observations on HES-related kidney disease with low to very low certainty evidence. The 40% IgG4/IgG ratio remains unvalidated and should not be used diagnostically. International prospective registries and individual patient data meta-analyses are needed to establish evidence-based management. These findings require prospective validation before clinical application.

## Highlights

- What is already known: HES affects multiple organs, but renal involvement is underrecognized. Diagnostic confusion with IgG4-related kidney disease poses clinical challenges.
- What this article adds: This systematic narrative review of 42 cases characterizes HES-related kidney disease pathology. Nine cases showed IgG4-RKD-like features with IgG4+/IgG+ ratios below 40%, but technical heterogeneity limits clinical applicability.
- How this impacts management: Clinicians should suspect renal involvement in HES patients with unexplained kidney dysfunction. The 40% IgG4/IgG ratio should not be used diagnostically. Biologic therapy evidence is limited to case reports with probable publication bias.

## Introduction

Hypereosinophilic syndrome (HES) comprises disorders characterized by sustained eosinophilia (absolute eosinophil count ≥1, 500/μL for ≥6 months) with evidence of target-organ damage (1). While cardiac, dermatologic, and pulmonary manifestations are well-recognized, renal involvement occurs in 5-10% of patients (2) and carries substantial morbidity. The true incidence likely exceeds these estimates, as subclinical presentations frequently escape detection without systematic renal evaluation.

The pathophysiology involves eosinophil degranulation with release of cytotoxic proteins including major basic protein, eosinophil peroxidase, and eosinophil cationic protein (3), which directly injure tissue parenchyma and promote fibrogenesis. However, the specific clinicopathologic patterns of renal involvement remain inadequately defined, complicating clinical recognition and management.

A critical diagnostic challenge lies in distinguishing HES-related renal lesions from IgG4-RKD. Both conditions can present with tubulointerstitial nephritis, storiform fibrosis, and elevated serum IgG4 levels (4, 5). Misdiagnosis carries significant therapeutic implications: IgG4-RKD typically responds to B-cell depletion with rituximab, whereas HES requires eosinophil-directed therapy. Recent case observations suggest IgG4+/IgG+ ratios below 40% in HES (6–8), but these findings derive from small, uncontrolled series with significant technical heterogeneity. The 40% threshold, derived from the 2019 ACR/EULAR IgG4-related disease classification criteria (9), has not been prospectively validated in HES populations.

The therapeutic landscape has evolved with anti-IL-5 (mepolizumab) and anti-IL-5Rα (benralizumab) biologics (10, 11). The phase 3 NATRON study demonstrated that benralizumab significantly delayed time to HES flare versus placebo (12). However, this pivotal trial excluded patients with eGFR <30 mL/min/1.73m² or dialysis dependence, leaving a critical evidence gap. Furthermore, the standard treatment for myeloproliferative HES with FIP1L1-PDGFRA fusion is imatinib, yet its specific efficacy in renal involvement remains poorly documented.

Given these uncertainties, we conducted this PROSPERO-registered systematic review (CRD420261307200) with a specific methodological choice: inclusion was restricted to cases with histopathologic confirmation of renal involvement. This decision requires explicit justification. While clinical suspicion of HES-related kidney disease can be established without biopsy, the phenotypic spectrum remains poorly defined because clinical syndromes (acute kidney injury, proteinuria) are nonspecific and may reflect multiple concurrent pathologies. Histopathologic confirmation provides: (1) definitive attribution of renal injury to HES rather than alternative etiologies; (2) characterization of specific pathologic patterns that may guide therapy; and (3) objective assessment of IgG4-related histologic features. This approach prioritizes phenotypic clarity over generalizability, acknowledging that milder or rapidly responding cases may be underrepresented.

The review aims to: (1) characterize the clinicopathologic spectrum of histologically confirmed HES-related kidney disease; (2) examine diagnostic overlap with IgG4-RKD; and (3) appraise the current evidence base for therapeutic strategies. All findings derive from uncontrolled case reports with very low certainty of evidence and require prospective validation before clinical application.

## Methods

### Study design and scope

This systematic review followed a PROSPERO-registered protocol (CRD420261307200, registered February 12, 2026) and employed systematic search methods for comprehensive case identification. The limited evidence (42 uncontrolled case reports spanning 40 years), high clinical and methodological heterogeneity, and absence of controlled studies precluded quantitative synthesis. Therefore, narrative synthesis is presented following Synthesis Without Meta-analysis (SWiM) guidelines (13).

Reporting follows PRISMA 2020 guidelines (14). Ethical approval was not required as only published, de-identified data were analyzed.

### Search strategy

PubMed, Embase, Cochrane Library, Web of Science, Scopus, and China National Knowledge Infrastructure (CNKI) were searched from inception to February 27, 2026. The search strategy combined MeSH and free-text terms for “hypereosinophilic syndrome, ““eosinophilia, ““kidney diseases, ““nephritis, “ and “renal insufficiency.” For IgG4-RKD comparative analysis, additional searches included “IgG4-related kidney disease, ““IgG4/IgG ratio, “ and “tubulointerstitial nephritis.” No language restrictions were applied initially; however, only English and Chinese articles could be fully assessed, potentially introducing language bias. Complete search strategies are provided in **Supplementary Table 1**.

### Inclusion and exclusion criteria

Cases were included if they met: (1) 2010 Simon criteria for HES¹; (2) histopathologic confirmation of renal involvement; and (3) availability of detailed clinicopathologic data. Exclusion criteria included secondary eosinophilia, eosinophilic granulomatosis with polyangiitis (EGPA) as primary diagnosis, studies lacking histopathologic correlation, review articles, editorials, conference abstracts, non-human studies, and duplicates.

The restriction to histopathologically confirmed cases improves phenotypic specificity but introduces substantial selection bias. This strategy may particularly underestimate milder cases, non-biopsied cases, or cases that improved after empirical clinical treatment. The resulting pathologic spectrum may not reflect the true distribution of renal involvement in unselected HES populations.

### Study selection and data extraction

Two reviewers independently screened records using Covidence software (inter-rater agreement Cohen’s κ = 0.89). Discrepancies were resolved by a third reviewer. Data extraction included demographics, HES subtype, renal presentation, histopathologic findings, immunohistochemistry results (IgG4+ plasma cell count, IgG4+/IgG+ ratio, antibody clone where reported), molecular testing (FIP1L1-PDGFRA fusion status where available), treatment, and outcomes. Extra-renal organ involvement and their response to therapy were specifically documented.

### Quality assessment and certainty of evidence

Study quality was assessed using the Joanna Briggs Institute Critical Appraisal Checklist for Case Reports (15). Studies scoring ≥5 of 8 were considered high quality. Using GRADE principles (16), all outcomes were rated very low certainty. This rating reflects: (1) risk of bias inherent in uncontrolled case reports with selective reporting; (2) inconsistency due to heterogeneous outcome definitions and treatment protocols; (3) indirectness as most evidence derives from adult populations without controlled comparisons; and (4) strong suspicion of publication bias (no treatment failures reported for biologics or imatinib). The critical instability concerns therapeutic conclusions: reported response rates likely overestimate true efficacy due to selective publication of positive outcomes.

## Results

### Study selection and characteristics

The initial search identified 2, 247 records. After removing duplicates, 1, 893 records were excluded at title/abstract screening. Full-text assessment of 354 articles led to exclusion of 317; 37 studies (42 cases) met inclusion criteria (**Supplementary Figure 1**). Publication years ranged from 1985 to 2026, with increased reporting since 2018. Quality assessment identified 28 high-quality and 14 moderate-quality case reports. Cases originated from 16 countries, predominantly Europe and Asia. No pediatric cases were identified, likely reflecting the adult predominance of HES and potential underrecognition in pediatric populations.

### Clinicopathologic spectrum

**Table 1** summarizes the pathologic patterns observed.

**Table 1:** Clinicopathologic patterns in HES-related kidney disease (N = 42).

Pathologic pattern	N (%)	Median age (y)	Key clinical features	HES subtype aAssociation	Treatment response	Long-term outcome
Acute EIN	23 (54.8)	48	AKI, fever (72%), rash (48%)	Lymphocytic (71%), idiopathic (22%), myeloproliferative (7%)	65% renal recovery with steroids	78% complete/partial recovery; 22% CKD
TMA	8 (19.0)	51	Severe HTN, nephrotic proteinuria (62.5%), dialysis (37.5%)	Myeloproliferative (87.5%), FIP1L1-PDGFRA (62.5%)	Limited data	37.5% ESRD
Chronic interstitial fibrosis	5 (11.9)	56	Slowly progressive CKD	Delayed diagnosis (median 22 mo)	Poor steroid response	80% ESRD
Glomerular lesions	6 (14.3)	44	Nephrotic syndrome, proteinuria	Idiopathic (67%), lymphocytic (33%)	Variable (MCD good, MN variable)	50% complete remission, 33% partial

EIN, eosinophilic interstitial nephritis; TMA, thrombotic microangiopathy; AKI, acute kidney injury; HTN, hypertension; CKD, chronic kidney disease; ESRD, end-stage renal disease; MCD, minimal change disease; MN, membranous nephropathy.

Critical Warning: All treatment response and outcome data derive from uncontrolled case reports with high publication bias. These figures should not be interpreted as evidence of treatment efficacy. No controlled studies exist.

Acute eosinophilic interstitial nephritis (EIN) was most frequent (54.8%, 23/42), characterized by dense eosinophilic infiltrates exceeding 20 cells per high-power field (median 48/HPF, range 24-156), often with Charcot-Leyden crystals and tubular necrosis. Clinical presentations included acute kidney injury, fever (72%), and rash (48%).

Thrombotic microangiopathy (TMA) occurred in 19.0% (8/42), predominantly in myeloproliferative HES (87.5%). Among these 8 TMA cases, FIP1L1-PDGFRA fusion testing was performed in 6 cases, with 5 positive (83.3% of tested cases, 62.5% of all TMA cases). These cases presented with severe hypertension, nephrotic-range proteinuria (62.5%), and rapid progression to dialysis (37.5%).

Chronic interstitial fibrosis (11.9%, 5/42) developed in patients with delayed diagnosis (median HES duration 22 months), showing extensive tubular atrophy and sparse eosinophils (median 4/HPF). These cases progressed to end-stage renal disease despite treatment.

Glomerular lesions (14.3%, 6/42) included membranous nephropathy (n=3), minimal change disease (n=2), and focal segmental glomerulosclerosis (n=1).

### Extra-renal organ involvement and treatment response

**Table 2** summarizes extra-renal manifestations and their evolution following therapy. Cardiac involvement was most common (45.2%, 19/42), presenting as eosinophilic myocarditis or restrictive cardiomyopathy. Dermatologic manifestations occurred in 38.1% (16/42), predominantly erythematous rashes and angioedema. Pulmonary involvement was reported in 28.6% (12/42), including eosinophilic pneumonia and pleural effusions. Gastrointestinal and neurologic involvement were less frequent (14.3% and 9.5%, respectively).

**Table 2:** Extra-renal organ involvement and post-treatment evolution (N = 42).

Organ system	N (%)	Common manifestations	Response to corticosteroids	Response to biologics
Cardiac	19 (45.2)	Myocarditis, restrictive cardiomyopathy	58% (11/19) improved	Limited data
Dermatologic	16 (38.1)	Erythematous rash, angioedema, pruritus	75% (12/16) resolved	Limited data
Pulmonary	12 (28.6)	Eosinophilic pneumonia, pleural effusion	67% (8/12) cleared	Limited data
Gastrointestinal	6 (14.3)	Eosinophilic gastroenteritis, abdominal pain	50% (3/6) improved	Limited data
Neurologic	4 (9.5)	Peripheral neuropathy, encephalopathy	25% (1/4) improved	Limited data

Response rates derive from uncontrolled case reports and likely overestimate true efficacy due to publication bias.

Following corticosteroid therapy, extra-renal manifestations showed variable response: cardiac symptoms improved in 58% (11/19) of cases, dermatologic lesions resolved in 75% (12/16), and pulmonary abnormalities cleared in 67% (8/12). However, these observations are confounded by disease severity, treatment timing, and reporting bias. Complete extra-renal organ involvement data are provided in **Supplementary Table 3**.

### Diagnostic overlap with IgG4-RKD

Nine cases (21.4%) presented features overlapping with IgG4-RKD: tubulointerstitial nephritis with storiform fibrosis, obliterative phlebitis, and elevated serum IgG4 (median 285 mg/dL, range 142-412). All demonstrated peripheral eosinophilia >1, 500/μL (median 4.8 × 10^9^/L) and extra-renal eosinophilic organ involvement. #The substantial technical heterogeneity in IgG4 immunohistochemistry across the nine overlapping cases is summarized in **Table 3**.

**Table 3:** Technical heterogeneity in IgG4 immunohistochemistry across included studies

Technical element	Variability observed	Clinical implication
Antibody clones	HP6025, G183-1, EPR3658, unspecified	Different clones have variable specificity for IgG4 subclasses
Staining protocols	Variable fixation, antigen retrieval, detection systems	Affects staining intensity and background signal
Quantification methods	Cutoffs: >10 to >50 cells/HPF; absolute counts vs semi-quantitative	Prevents standardization across centers
Ratio calculation	Denominators varied (total IgG+ cells vs total plasma cells)	Results not comparable across studies

Critical Technical Heterogeneity in IgG4 Immunohistochemistry:

Immunohistochemical staining for IgG and IgG4 in these nine cases revealed substantial technical heterogeneity that fundamentally limits comparability and clinical application:

No centralized pathology review was available. These technical variations introduce significant measurement bias and underscore why the IgG4+/IgG+ ratio should not be used as a standalone diagnostic criterion.

The IgG4+/IgG+ ratio was below 40% in all nine cases (median 21%, IQR 14-30%). In contrast, published IgG4-RKD cohorts report median ratios of 55-70% (range 42-85%) (17–19). This observation is presented for hypothesis generation only; it requires validation with standardized protocols before any clinical application.

Three of these nine cases were initially misdiagnosed as IgG4-RKD and received rituximab without response, highlighting the clinical consequences of misclassification.

### Pathologic observations regarding IgG4-RKD differentiation

Beyond IgG4/IgG ratios, several histopathologic features observed in case reports may provide clues for differentiation:

Eosinophil infiltration pattern: HES-related EIN typically shows dense, diffuse eosinophilic infiltrates (often >20/HPF, sometimes forming microabscesses) with abundant Charcot-Leyden crystals. In contrast, IgG4-RKD shows sparse, “sprinkled” eosinophils (usually <5/HPF) without microabscess formation.

Tubular injury: HES shows prominent tubular necrosis and acute tubular injury, whereas IgG4-RKD demonstrates tubular atrophy with storiform fibrosis but relatively preserved tubular architecture early in disease.

Vascular involvement: Obliterative phlebitis in IgG4-RKD involves veins with lymphoplasmacytic infiltrate and fibrosis; HES may show eosinophilic vasculitis or thrombotic microangiopathy in myeloproliferative variants.

Immunofluorescence: IgG4-RKD may show granular IgG4 deposition along tubular basement membranes; HES typically shows negative or non-specific staining.

However, these observations derive from case reports without systematic comparative pathology, and inter-observer reliability is unknown. They should not be interpreted as definitive diagnostic criteria.

### Treatment outcomes by pathology and HES subtype

**Table 4** summarizes treatment outcomes organized by both renal pathology and HES subtype to address the clinical question of whether treatment decisions should be based on hematologic subtype, kidney pathology, or both.

**Table 4:** Treatment outcomes by renal pathology and HES subtype (N = 42).

Renal pathology	HES subtype	N	First-line therapy	Response	Key limitations
Acute EIN	Lymphocytic	16	Corticosteroids	69% renal recovery	Uncontrolled; 44% relapse on taper
Acute EIN	Idiopathic	5	Corticosteroids	60% renal recovery	Small sample; variable follow-up
Acute EIN	Myeloproliferative	2	Imatinib ± steroids	50% renal recovery	Very limited data
TMA	Myeloproliferative (FIP1L1-PDGFRA+)	5	Imatinib	40% renal response, 40% stabilization, 20% ESRD	Hematologic response universal; renal response variable
TMA	Myeloproliferative (FIP1L1-PDGFRA-)	3	Corticosteroids ± imatinib trial	Limited data	No systematic TKI trial data
Chronic fibrosis	Various	5	Corticosteroids	Poor response (20% stabilization)	Irreversible damage at presentation
Glomerular lesions	Idiopathic	4	Corticosteroids	Variable (MCD good, MN moderate)	Pathology-specific response
Glomerular lesions	Lymphocytic	2	Corticosteroids	50% response	Very limited data

EIN, eosinophilic interstitial nephritis; TMA, thrombotic microangiopathy; MCD, minimal change disease; MN, membranous nephropathy; TKI, tyrosine kinase inhibitor.

Critical Warnings: Outcome definitions varied substantially across reports. No control group existed. The complete absence of documented treatment failures strongly suggests publication bias. These data cannot establish efficacy or safety. The NATRON trial excluded patients with eGFR <30 mL/min/1.73m²; no randomized data exist for renally impaired HES patients.

Corticosteroids: Remained first-line therapy (85.7%), with reported renal recovery in 65.2% of acute EIN cases when treated within 4 weeks. However, 47.8% relapsed during taper. These observations are uncontrolled and confounded by disease severity, treatment timing, and publication bias.

Imatinib for Myeloproliferative HES: Among the 5 FIP1L1-PDGFRA-positive TMA cases, 4 received imatinib (100–400 mg/day). Reported outcomes included hematologic remission (eosinophil normalization) in all 4, with renal response (eGFR improvement >25%) in 2 cases, stabilization in 1, and progression to ESRD despite hematologic response in 1. One case received upfront combination therapy with corticosteroids and imatinib. These limited data suggest that imatinib achieves hematologic control but renal recovery is variable and depends on baseline kidney function and chronicity of lesions. Notably, the NATRON trial excluded these patients, leaving a critical evidence gap for tyrosine kinase inhibitor therapy in HES-related kidney disease.

Conventional Immunosuppressants: Mycophenolate mofetil was used in 3 steroid-dependent cases (1 lymphocytic, 2 idiopathic) with reported steroid-sparing effect; cyclophosphamide was used in 1 refractory lymphocytic HES with partial response. Data are insufficient to assess efficacy.

**Figure 1:**
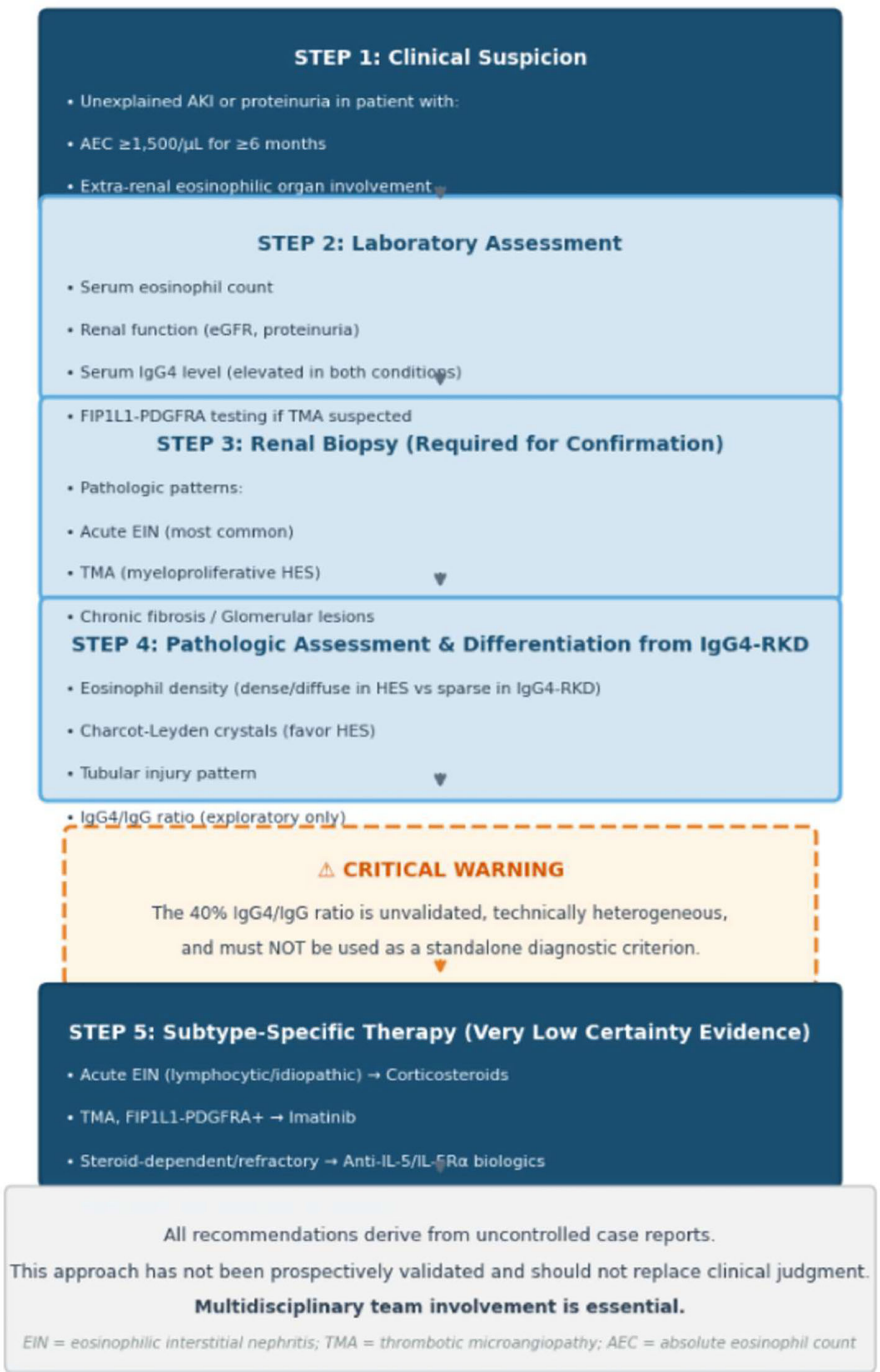
Proposed clinical approach to suspected HES-related kidney disease (unvalidated, evidence-limited). This exploratory algorithm integrates clinical suspicion, pathologic interpretation (with explicit warnings about the unvalidated IgG4/IgG ratio), and treatment considerations. All recommendations derive from very low certainty evidence (uncontrolled case reports). The 40% IgG4/IgG ratio threshold is exploratory only, technically heterogeneous, and must not be used as a diagnostic criterion. Multidisciplinary team involvement is essential. This approach has not been prospectively validated and should not replace clinical judgment.

**Figure 2:**
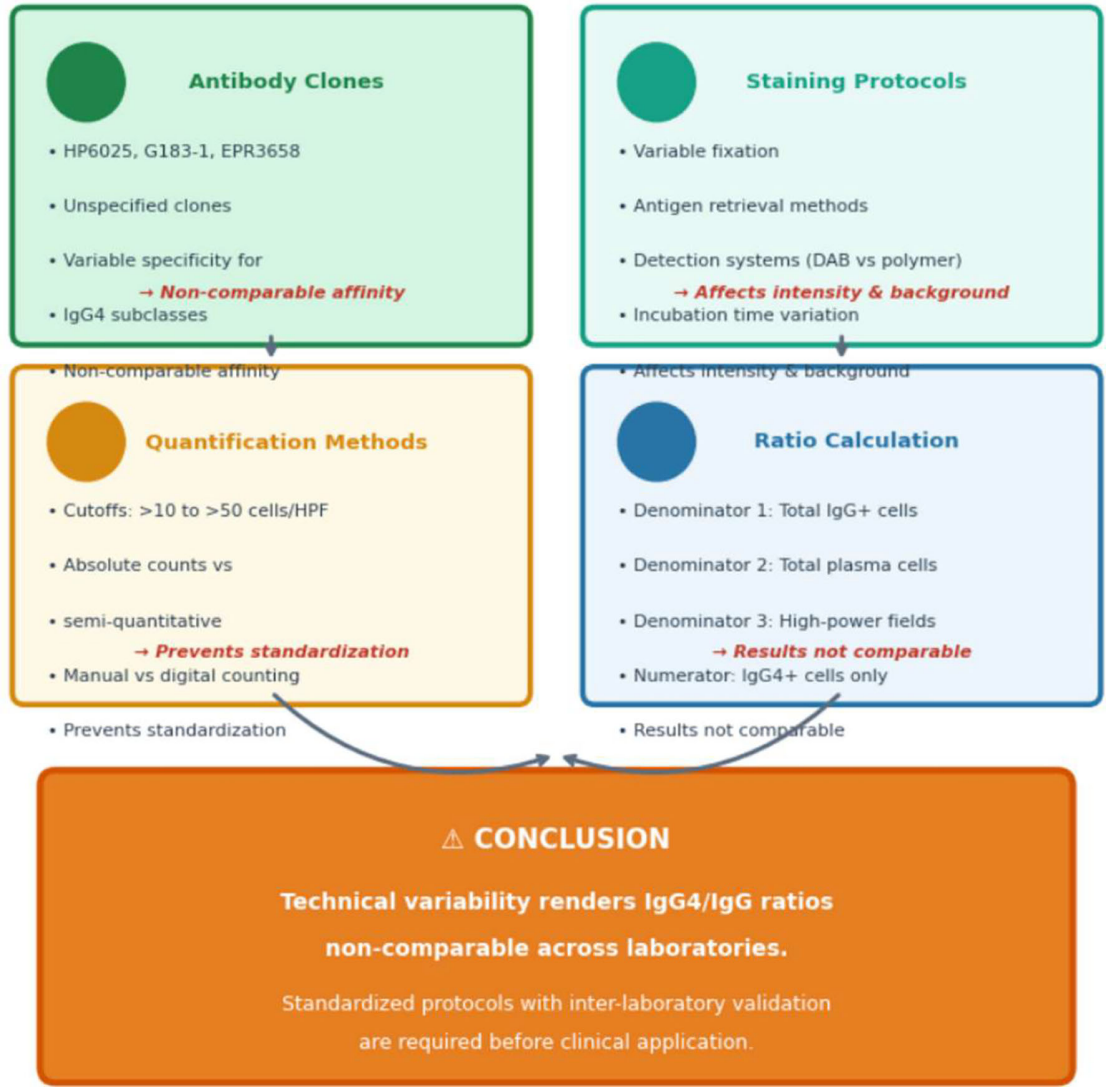
Schematic illustration of technical heterogeneity sources in IgG4 immunohistochemistry. This figure provides a visual summary of the key sources of variability in IgG4/IgG ratio assessment across laboratories: **(A)** antibody clone selection affecting specificity; **(B)** pre-analytical factors including fixation and antigen retrieval; **(C)** quantification methods showing variable cutoffs and counting approaches; and **(D)** ratio calculation demonstrating different denominator options. The schematic emphasizes why standardization is prerequisite to any clinical application of IgG4/IgG ratios.

Anti-IL-5/IL-5Rα Biologics: Fourteen cases treated with biologics were reported since 2020. Critically, no treatment failures were documented, strongly suggestive of publication bias. **Table 4** summarizes these cases with explicit caveats. A proposed clinical approach to suspected HES-related kidney disease is outlined in **Figure 1**. Key sources of technical heterogeneity in IgG4 immunohistochemistry are summarized schematically in **Figure 2**.

## Discussion

This systematic review with narrative synthesis of 42 uncontrolled case reports provides hypothesis-generating observations on HES-related kidney disease with very low certainty of evidence. The predominance of acute EIN aligns with established cytotoxic mechanisms of eosinophil granule proteins. The association of TMA with myeloproliferative HES highlights the role of eosinophil-derived mediators in endothelial injury (20–22).

### Critical methodological limitations

Several limitations require explicit emphasis. First, the restriction to histopathologically confirmed cases, while improving phenotypic specificity, introduces substantial selection bias. This strategy likely underestimates milder cases that responded to empirical therapy without biopsy, cases where biopsy was contraindicated due to severe renal dysfunction, and cases with isolated extra-renal manifestations. The resulting pathologic spectrum may not reflect the true distribution of renal involvement in unselected HES populations.

Second, publication bias is strongly suspected. The complete absence of documented treatment failures for biologics (14 cases) and imatinib in FIP1L1-PDGFRA-positive cases (4 cases) is statistically implausible if these therapies have moderate efficacy. This selective reporting likely overestimates true response rates and obscures adverse event profiles.

Third, technical heterogeneity in IgG4 immunohistochemistry fundamentally limits the clinical applicability of the IgG4+/IgG+ ratio observation. Varying antibody clones, staining protocols, quantification methods, and ratio denominators across centers produce non-comparable results. Without standardized methodologies and inter-laboratory validation, the 40% threshold cannot be used diagnostically.

Fourth, language bias may have excluded relevant Japanese literature, where HES recognition is high. Finally, inconsistent outcome definitions preclude quantitative synthesis; “improvement” was variously defined as eGFR increase, dialysis independence, proteinuria reduction, or steroid-sparing effect.

### Immunologic mechanisms of TMA in HES

The strong association between TMA and myeloproliferative HES (particularly FIP1L1-PDGFRA-positive cases) warrants deeper immunologic consideration. Eosinophils release major basic protein and eosinophil peroxidase, which directly damage endothelial cells. Additionally, FIP1L1-PDGFRA fusion drives constitutive PDGFRA signaling, promoting platelet activation and thrombosis. The combination of eosinophil-mediated endothelial injury and hypercoagulability creates a “double hit” mechanism for TMA development. This pathophysiology explains why TMA in HES may be less responsive to standard TMA therapies (e.g., plasma exchange) compared to complement-mediated or ADAMTS13-deficient TMA, and why targeted therapy with imatinib (addressing the clonal eosinophil population) may be essential. Recent comprehensive reviews of eosinophil-associated diseases support these mechanisms (23, 24).

### IgG4 elevation in HES: Th2 polarization and class switching

The observation that 21.4% of HES cases exhibited elevated IgG4 and IgG4-RKD-like features raises important immunologic questions. HES is characterized by Th2 polarization, with elevated IL-4, IL-5, and IL-13. IL-4 and IL-13 are known drivers of IgG4 class switching through induction of activation-induced cytidine deaminase (AID) and specific targeting of the IgG4 Cγ4 gene segment (25). Thus, elevated IgG4 in HES may reflect the underlying Th2 milieu rather than true IgG4-RKD. This hypothesis is supported by the observed IgG4+/IgG+ ratios below 40% (compared to 55-70% in IgG4-RKD), suggesting polyclonal IgG4 elevation rather than the oligoclonal, antigen-driven expansion characteristic of IgG4-RKD. Recent updates on the classification and management of eosinophilic disorders provide additional context for understanding these immunologic overlaps (26, 27).

### Critical diagnostic warning: the 40% IgG4/IgG ratio

A central finding is that 21.4% of cases exhibited features overlapping with IgG4-RKD, and all demonstrated IgG4+/IgG+ ratios below 40% (17–19). However, this observation must not be misinterpreted as a validated diagnostic threshold for several reasons:

Technical heterogeneity: Varying antibody clones, staining protocols, and quantification methods across centers produce non-comparable results.

No prospective validation: The 40% threshold derives from IgG4-RD classification criteria and has never been validated in HES populations.

No centralized pathology review: Inter-laboratory variability remains uncharacterized.

Therefore, the IgG4+/IgG+ ratio should not be used as a standalone diagnostic criterion in clinical practice. Its only role, if any, is as one reference indicator among many in expert multidisciplinary settings, and only after local laboratory validation against known controls. Comparative studies of EGPA and IgG4-related disease highlight the importance of careful histopathologic differentiation in eosinophilic disorders (28).

### Considerations for differential diagnosis

When evaluating suspected HES-related kidney disease with IgG4-RKD overlap features, we suggest considering the following clinical and pathologic clues, with explicit acknowledgment that these derive from case reports rather than systematic comparative studies:

Clinical context: Peripheral eosinophil count and duration (>1, 500/μL for >6 months), extra-renal eosinophilic organ involvement (cardiac, pulmonary, dermatologic), and serum IgG4 level (elevated in both conditions, generally higher in IgG4-RKD).

Pathologic assessment: Eosinophil density and distribution (dense/diffuse in HES vs sparse/sprinkled in IgG4-RKD), presence of Charcot-Leyden crystals (favor HES), pattern of fibrosis (storiform with obliterative phlebitis in IgG4-RKD; more variable in HES), and tubular injury pattern (acute necrosis in HES vs atrophy with relative preservation in IgG4-RKD).

Immunohistochemistry: IgG4+ plasma cell count and IgG4+/IgG+ ratio may provide exploratory clues, but technical heterogeneity limits comparability.

Therapeutic response: Response to corticosteroids (typically rapid in HES, variable in IgG4-RKD) and failure of rituximab (suggests HES rather than IgG4-RKD).

Recent updates on IgG4-related disease provide additional guidance on diagnostic criteria and classification (29).

### Evidence limitations and therapeutic decision-making

The therapeutic evidence base is severely limited. For acute EIN in lymphocytic or idiopathic HES, corticosteroids remain empiric first-line therapy based on rapid eosinophil cytoreduction, though relapse rates are high during taper. For myeloproliferative HES with FIP1L1-PDGFRA fusion, imatinib is standard of care for hematologic control, but renal recovery is variable and early treatment may prevent irreversible TMA. For steroid-dependent or refractory cases, anti-IL-5/IL-5Rα biologics show promise but evidence is limited to case reports with uniform reporting of improvement, strongly suggesting publication bias. The NATRON trial excluded patients with eGFR <30 mL/min/1.73m², leaving a critical evidence gap for the most severely affected patients.

Clinicians should not interpret the reported 100% “response rate” for biologics as evidence of efficacy. These data cannot establish effectiveness or safety. Use should be restricted to selected steroid-dependent cases under multidisciplinary guidance with explicit patient discussion of evidence limitations.

Clinical management of HES requires careful consideration of subtype-specific approaches (30).

### Future research priorities

International prospective registry (highest priority): Given the rarity of HES-related kidney disease, international collaboration is essential to accumulate sufficient cases for meaningful analysis.

Individual patient data (IPD) meta-analysis: As case reports accumulate, IPD meta-analysis could address heterogeneity through standardized re-analysis of raw data, adjusting for confounders unavailable in aggregate reporting.

Standardized pathology consensus: Development of standardized immunohistochemistry protocols (antibody clones, staining protocols, cutoffs) with inter-laboratory validation.

Randomized controlled trials including eGFR<30 patients: Current trials exclude the most severely affected patients; dedicated studies in renally impaired populations are urgently needed.

Prospective validation of diagnostic observations: The proposed clinical and pathologic clues require prospective testing in consecutive biopsy series.

Pediatric studies: HES in children may have different clinicopathologic features; dedicated pediatric series are needed.

## Conclusions

This PROSPERO-registered systematic review with narrative synthesis of 42 uncontrolled case reports provides hypothesis-generating observations on HES-related kidney disease with very low certainty evidence. Acute eosinophilic interstitial nephritis is the predominant reported pattern. Diagnostic ambiguity with IgG4-RKD exists; however, the 40% IgG4/IgG ratio threshold remains unvalidated, technically heterogeneous, and must not be used as a diagnostic criterion. Evidence for anti-IL-5/IL-5Rα therapies is limited to case reports with uniform reporting of improvement, strongly suggesting publication bias, and no RCT data exist for renally impaired patients. For myeloproliferative HES with FIP1L1-PDGFRA fusion, imatinib remains standard of care but renal outcomes are variable. International prospective registries, individual patient data meta-analyses, and trials including patients with eGFR <30 mL/min/1.73m² are urgently needed. These findings are hypothesis-generating only and require prospective validation before clinical application.
