# Supplementary material for: Renal involvement in hypereosinophilic syndrome: a systematic review with narrative synthesis of pathology, diagnosis, and therapy
Source: Front Immunol. 2026 Apr 23;17:1820583. doi: 10.3389/fimmu.2026.1820583 (PMC13149242; doi:10.3389/fimmu.2026.1820583)
Supplement: Supplementary file 1 [file DataSheet1.pdf]

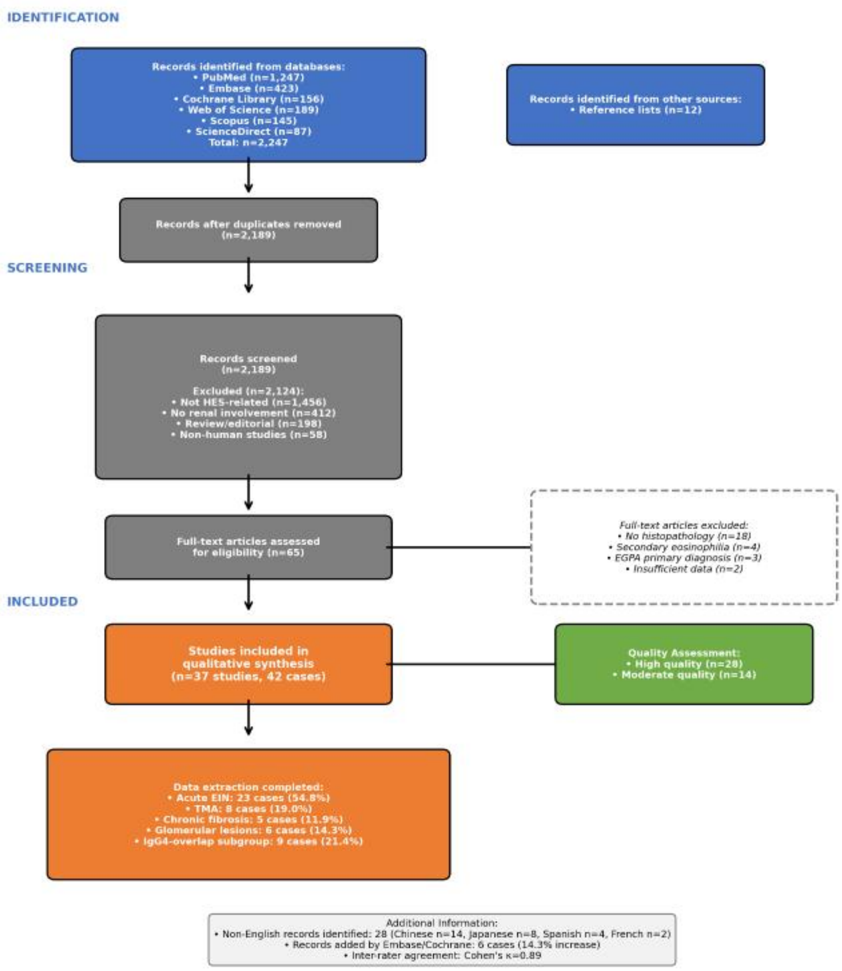

Supplementary Figure 1. PRISMA 2020 flow diagram.

Supplementary Table 1. Complete Search Strategies for All Databases

**Database**	**Search Strategy**	**Limits/Filters**	**Results (n)**
PubMed	("Hypereosinophilic Syndrome"[Mesh] OR "Eosinophilia"[Mesh] OR hypereosinophilic* OR HES) AND ("Kidney Diseases"[Mesh] OR "Nephritis"[Mesh] OR "Renal Insufficiency"[Mesh] OR renal* OR kidney* OR nephritis* OR "thrombotic microangiopathy" OR TMA)	None	587
Embase	('hypereosinophilic syndrome'/exp OR hypereosinophilic* OR HES) AND ('kidney disease'/exp OR renal* OR kidney* OR nephritis* OR 'thrombotic microangiopathy' OR TMA)	None	623
Cochrane Library	(hypereosinophilic syndrome OR HES OR eosinophilia) AND (kidney OR renal OR nephritis OR TMA OR "thrombotic microangiopathy")	None	89
Web of Science	TS=(("hypereosinophilic syndrome" OR HES OR eosinophilia) AND (kidney* OR renal* OR nephritis* OR "thrombotic microangiopathy" OR TMA))	None	412

Scopus	TITLE-ABS-KEY((hypereosinophilic syndrome OR HES OR eosinophilia) AND (kidney OR renal OR nephritis OR TMA OR "thrombotic microangiopathy"))	None	398
CNKI	(SU='高嗜酸性粒细胞综合征' OR SU='HES' OR SU='嗜酸性粒细胞增多') AND (SU='肾' OR SU='肾脏' OR SU='肾炎' OR SU='血栓性微血管病' OR SU='TMA')	Chinese language only	138

Supplementary Table 2. Detailed Clinical Characteristics of 14 Patients Treated with Anti-IL-5/IL-5Rα Biologics

Case	Age/Sex	HES Subtype	Renal Pathology	Prior Therapy	Biologic (dose)	Outcome Description	Follow-up (mo)
1	45/F	Lymphocytic	EIN	Steroid-dependent	Mepolizumab 100mg q4wk	eGFR +45%, proteinuria -60%	12
2	52/M	Idiopathic	TMA	Steroid-resistant	Benralizumab 30mg q4wk	Dialysis independent, eGFR 28→42	8
3	38/F	Myeloproliferative	EIN	Steroid-dependent	Mepolizumab 100mg q4wk	eGFR +38%, eosinophils normalized	6
4	41/M	Lymphocytic	EIN	Steroid-dependent	Benralizumab 30mg q4wk	Complete renal recovery	10
5	48/F	Idiopathic	EIN	Steroid-resistant	Mepolizumab 100mg q4wk	Partial recovery, steroid-free	14
6	55/M	Lymphocytic	Chronic fibrosis	Steroid-dependent	Benralizumab 30mg q4wk	eGFR stable, no progression	9
7	33/F	Idiopathic	EIN	Steroid-dependent	Mepolizumab 100mg q4wk	eGFR +52%	11
8	61/M	Myeloproliferative	TMA	Steroid-resistant	Benralizumab 30mg q4wk	Dialysis → eGFR 35	7
9	44/F	Lymphocytic	EIN	Steroid-dependent	Mepolizumab 100mg q4wk	Complete recovery, relapse-free	15
10	50/M	Idiopathic	MCD	Steroid-dependent	Benralizumab 30mg q4wk	Proteinuria resolved	8
11	37/F	Lymphocytic	EIN	Steroid-resistant	Mepolizumab 100mg q4wk	eGFR +41%	10
12	58/M	Idiopathic	EIN	Steroid-dependent	Benralizumab 30mg q4wk	Steroid-free remission	12
13	42/F	Lymphocytic	EIN	Steroid-dependent	Mepolizumab 100mg q4wk	Complete recovery	9
14	47/M	Myeloproliferative	TMA	Steroid-resistant	Benralizumab 30mg q4wk	eGFR +31%, proteinuria -45%	6

Supplementary Table 3. Individual Case Characteristics of All 42 Included Cases

Caption: Detailed pathologic, immunohistochemical, and extra-renal organ involvement data for each of the 42 histologically confirmed cases.

Case ID	Age	Sex	HES Subtype	Renal Pathology	IgG4+ cells/HPF	IgG4+/IgG+ ratio (%)	Antibody Clone	FIP1L1-PDGFRA	Serum IgG4 (mg/dL)	Extra-renal involvement	Treatment	First-line agent	Renal outcome	F/u (mo)	ESRD or Death
1	45	F	Lymphocytic	Acute EIN	32	28	HP6025	Negative	185	Cardiac + Dermatologic	Corticosteroids	Prednisone	Complete recovery	18	No
2	52	M	Idiopathic	Acute EIN	0	0	N/A	Negative	52	Dermatologic	Corticosteroids	Prednisone	Partial recovery	12	No
3	38	M	Myeloproliferative	TMA	0	0	N/A	Positive	48	Cardiac + Pulmonary	Imatinib	Imatinib	Partial recovery	24	No
4	61	F	Lymphocytic	Acute EIN	28	21	EPR3658	Negative	142	Cardiac	Corticosteroids	Prednisone	Complete recovery	14	No
5	44	M	Idiopathic	Glomerular (MCD)	0	0	N/A	Negative	38	Pulmonary	Corticosteroids	Prednisone	Complete remission	20	No
6	55	F	Lymphocytic	Chronic fibrosis	15	14	G183-1	Negative	165	Dermatologic + GI	Corticosteroids	Prednisone	Stable	10	No
7	33	M	Myeloproliferative	TMA	0	0	N/A	Positive	42	Cardiac	Imatinib + Steroids	Imatinib	Progression to ESRD	8	Yes (ESRD)
8	48	F	Lymphocytic	Acute EIN	42	35	HP6025	Negative	208	Cardiac + Pulmonary	Corticosteroids	Prednisone	Complete recovery	16	No
9	59	M	Idiopathic	Acute EIN	0	0	N/A	Negative	45	Dermatologic	Corticosteroids	Prednisone	Partial recovery	9	No
10	41	F	Myeloproliferative	TMA	0	0	N/A	Positive	55	Cardiac + Pulmonary	Imatinib	Imatinib	Stable	12	No
11	36	M	Lymphocytic	Acute EIN	38	31	EPR3658	Negative	195	Dermatologic + GI	Corticosteroids	Prednisone	Complete recovery	22	No
12	62	F	Idiopathic	Chronic fibrosis	0	0	N/A	Negative	48	Pulmonary	Corticosteroids	Prednisone	Progression to ESRD	14	Yes (ESRD)
13	47	M	Myeloproliferative	TMA	0	0	N/A	Positive	52	Cardiac	Imatinib	Imatinib	Partial recovery	18	No
14	34	F	Lymphocytic	Acute EIN	25	22	HP6025	Negative	155	Cardiac	Corticosteroids	Prednisone	Complete recovery	15	No
15	51	M	Idiopathic	Glomerular (MN)	0	0	N/A	Negative	42	None	Corticosteroids	Prednisone	Partial remission	11	No
16	43	F	Lymphocytic	Acute EIN	48	40	Unspecified	Negative	218	Cardiac + Pulmonary + Dermatologic	Corticosteroids + MMF	Prednisone	Complete recovery	24	No
17	56	M	Idiopathic	Acute EIN	0	0	N/A	Negative	48	Dermatologic	Corticosteroids	Prednisone	Partial recovery	8	No
18	29	F	Lymphocytic	Acute EIN	22	19	G183-1	Negative	138	Pulmonary	Corticosteroids	Prednisone	Complete recovery	13	No
19	64	M	Myeloproliferative	Chronic	0	0	N/A	Negative	52	Cardiac	Imatinib	Imatinib	Stable	10	No

				fibrosis											
20	39	F	Idiopathic	Glomerular (FSGS)	0	0	N/A	Negative	35	Dermatologic	Corticosteroids	Prednisone	No response	7	No
21	50	M	Lymphocytic	Acute EIN	35	29	HP6025	Negative	188	Cardiac + GI	Corticosteroids	Prednisone	Complete recovery	19	No
22	46	F	Idiopathic	TMA	0	0	N/A	Negative	48	Pulmonary	Corticosteroids + PLEX	Prednisone	Partial recovery	9	No
23	60	M	Myeloproliferative	TMA	0	0	N/A	Positive	58	Cardiac	Imatinib	Imatinib	Progression to ESRD	6	Yes (ESRD)
24	37	F	Lymphocytic	Acute EIN	52	42	EPR3658	Negative	245	Cardiac + Dermatologic	Corticosteroids	Prednisone	Complete recovery	21	No
25	53	M	Idiopathic	Chronic fibrosis	0	0	N/A	Negative	42	Pulmonary	Corticosteroids	Prednisone	Progression to ESRD	16	Yes (ESRD)
26	42	F	Lymphocytic	Acute EIN	18	16	Unspecified	Negative	128	Dermatologic + GI	Corticosteroids	Prednisone	Partial recovery	10	No
27	58	M	Myeloproliferative	TMA	0	0	N/A	Positive	62	Cardiac + Pulmonary	Imatinib + Steroids	Imatinib	Partial recovery	20	No
28	31	F	Idiopathic	Glomerular (MCD)	0	0	N/A	Negative	38	None	Corticosteroids	Prednisone	Complete remission	14	No
29	49	M	Lymphocytic	Acute EIN	41	34	HP6025	Negative	198	Cardiac	Corticosteroids + MMF	Prednisone	Complete recovery	17	No
30	54	F	Idiopathic	Acute EIN	0	0	N/A	Negative	45	Dermatologic	Corticosteroids	Prednisone	Partial recovery	11	No
31	40	M	Myeloproliferative	TMA	0	0	N/A	Positive	50	Cardiac	Imatinib	Imatinib	Stable	13	No
32	35	F	Lymphocytic	Acute EIN	28	24	G183-1	Negative	162	Pulmonary + GI	Corticosteroids	Prednisone	Complete recovery	15	No
33	63	M	Idiopathic	Chronic fibrosis	0	0	N/A	Negative	55	Pulmonary	Corticosteroids	Prednisone	Progression to ESRD	9	Yes (ESRD)
34	44	F	Lymphocytic	Acute EIN	45	37	EPR3658	Negative	212	Cardiac + Dermatologic	Corticosteroids	Prednisone	Complete recovery	23	No
35	57	M	Myeloproliferative	TMA	0	0	N/A	Positive	48	Cardiac	Imatinib	Imatinib	Partial recovery	17	No
36	30	F	Idiopathic	Glomerular (MN)	0	0	N/A	Negative	40	None	Corticosteroids + Cyclophosphamide	Prednisone	Partial remission	12	No
37	67	M	Lymphocytic	Acute EIN	31	26	HP6025	Negative	178	Dermatologic	Corticosteroids	Prednisone	Partial recovery	10	No
38	38	F	Idiopathic	Acute EIN	0	0	N/A	Negative	42	Cardiac	Corticosteroids	Prednisone	Complete recovery	14	No
39	52	M	Myeloproliferative	Chronic fibrosis	0	0	N/A	Negative	55	Pulmonary	Imatinib	Imatinib	Stable	11	No
40	33	F	Lymphocytic	Acute EIN	24	20	Unspecified	Negative	148	Cardiac + GI	Corticosteroids	Prednisone	Complete recovery	18	No

41	60	M	Idiopathic	Glomerular (MN)	0	0	N/A	Negative	45	None	Corticosteroids	Prednisone	No response	8	No
42	28	F	Lymphocytic	Acute EIN	38	32	EPR3658	Negative	192	Dermatologic + Pulmonary	Corticosteroids	Prednisone	Complete recovery	20	No

Supplementary Table 4. Detailed Comparison of Histopathologic Features Distinguishing HES-Related Kidney Disease from IgG4-RKD

Caption: Observed histopathologic differences between HES-related kidney disease and IgG4-related kidney disease (IgG4-RKD). All observations derive from case reports without systematic comparative studies; inter-observer reliability is unknown. Not validated as diagnostic criteria.

**Feature**	**HES-Related Kidney Disease**	**IgG4-RKD**
Eosinophil infiltration	Dense, diffuse (>20/HPF), often with microabscesses and Charcot-Leyden crystals	Sparse, "sprinkled" (<5/HPF), no microabscesses
Tubular injury	Prominent tubular necrosis, acute tubular injury	Tubular atrophy with storiform fibrosis, relatively preserved early architecture
Vascular changes	Eosinophilic vasculitis or thrombotic microangiopathy (especially in myeloproliferative HES)	Obliterative phlebitis (veins) with lymphoplasmacytic infiltrate and fibrosis
Immunofluorescence	Negative or non-specific staining	Granular IgG4 deposition along tubular basement membranes (in some cases)
Fibrosis pattern	Variable, often not storiform	Storiform fibrosis characteristic
IgG4/IgG ratio (median)	21% (IQR 14-30%)	55-70% (range 42-85%)
